# Recent progress and perspectives of advanced Ni-based cathodes for aqueous alkaline Zn batteries

**DOI:** 10.3389/fchem.2024.1483867

**Published:** 2024-11-26

**Authors:** Yanfen Ma, Xin Song, Wenjing Hu, Jiawei Xiong, Pan Chu, Yanchen Fan, Biao Zhang, Hongyu Zhou, Chenguang Liu, Yi Zhao

**Affiliations:** ^1^ Petro China Shen Zhen: New Energy Research Institute, Shenzhen, China; ^2^ State Key Laboratory of Chemical Resource Engineering, Beijing Advanced Innovation Center for Soft Matter Science and Engineering, College of Chemistry, Beijing University of Chemical Technology, Beijing, China; ^3^ Mary Frances Early College of Education, The University of Georgia, Athens, GA, United States

**Keywords:** alkaline Zn batteries, Ni-based cathodes, energy storage mechanism, optimization strategies, challenges and perspectives

## Abstract

Rechargeable aqueous alkaline Zn-Ni batteries (AZNBs) are considered a potential contender for energy storage fields and portable devices due to their inherent safety, high output voltage, high theoretical capacity and environmental friendliness. Despite the facilitated development of AZNBs by many investigations, its practical application is still restricted by inadequate energy density, sluggish kinetics, and poor stability. Therefore, Ni-based cathodes with boosted redox chemistry and enhanced structural integrity is essential for the high-performance AZNBs. Herein, this review focus on critical bottlenecks and effective design strategies of the representative Ni-based cathode materials. Specifically, nanostructured optimization, defect engineering, ion doping, heterostructure regulation and ligand engineering have been employed from the fundamental aspects for high-energy and long-lifespan Ni-based cathodes. Finally, further exploration in failure mechanism, binder-free battery configurations, practical application scenarios, as well as battery recycling are considered as valuable directions for the future development of advanced AZNBs.

## 1 Introduction

Faced with the growing shortage of fossil fuels and the aggravation of environmental pollution, the development and utilization of new energy sources have gradually become a research focus (Molaiyan et al., 2024). However, the wind, solar and wave energy generally exhibit the disadvantages of intermittent operation, regional distribution, and immovably confined (Mahlia et al., 2014). Thus, reliable and safe energy storage systems are crucial for building a clean and reliable power grid (Fan et al., 2021; Zhao et al., 2020). Actually, lithium-ion batteries (LIBs) and supercapacitors have been the two major energy storage systems in many fields. As for supercapacitors, a lot of researches have been carried out to improve their electrochemical performance. For instance, a hexagonal-CoMn_2_O_4_ electrode synthesized by KOH mediated hydrothermally approach delivers a great capacitance (638.8 F g^−1^) and remarkable cyclic stability (85% capacity retention after 4000 charge-discharge cycles) (Ramachandran et al., 2022). Nevertheless, the energy density of supercapacitiors should be further efficiently raised to boost their large scale application. To date, lithium-ion batteries (LIBs) currently dominate the rechargeable battery market due to their high energy density and extended lifespan (Wang et al., 2016). Yet, the resource-constrained Li/Co materials and flammable organic electrolytes severely limit the further development of LIBs (Wang Y. et al., 2022). Consequently, it is urgent to develop a high-security and low-cost battery technology for large-scale energy storage systems (Konarov et al., 2018). Due to intrinsic safety, environmental friendliness, low cost, as well as high theoretical capacity (820 mAh g^−1^), aqueous Zn batteries are considered as one of the most promising alternatives or supplements for LIBs (Li L. et al., 2022; Chen R. et al., 2024; Yuan et al., 2023; Wang Q. et al., 2022). Currently, Mn-based oxides, V-based oxides, Ni-based hosts, prussian blue analogues, and some organic compounds have been employed as cathode materials for aqueous Zn batteries (Wang Y. et al., 2024; Yan et al., 2024; Chen X. et al., 2022; Zhao Y. et al., 2021). Notably, Zn anodes exhibit more negative redox potential of −1.26 V (vs. standard hydrogen electrode, SHE) in alkaline electrolytes than that of −0.76 V in mild/acid electrolytes (Li J. et al., 2018). Typical Ni-based cathodes such as nickel hydroxide (Ni(OH)_2_) exhibit the redox reaction of Ni(OH)_2_/NiOOH with the potential of 0.49 V (vs. SHE). Therefore, alkaline Zn-Ni batteries (AZNBs) are attracting intensive attentions in next-generation batteries owing to their higher output voltage (∼1.75 V) than that of mild aqueous Zn-Mn (∼1.35 V), Zn-V (∼0.6 V), and Zn-organic batteries (<1.2 V) (Chao et al., 2019; Abdalla et al., 2024; Yuan et al., 2022).

In recent years, a serious of successes have been reported for achieving high-utilization and long-life cathode materials in aqueous batteries (Jia et al., 2020). Specifically, Ni(OH)_2_, NiO, NiS_2_, and other Ni compounds are investigated as the Ni-based cathodes for aqueous AZNBs (Lee et al., 2016; Lu et al., 2015). Reportedly, Parjer’ group developed a Ni-3D Zn battery based on a Zn sponge anode, which demonstrates a specific energy equivalent to lithium-ion systems (Parker et al., 2014). Zhou et al. have prepared a Co-free Ni-based microsphere cathode, which is a cost-effective alternative to Zn-Ni battery (Zhou et al., 2020c). Yet, the development of Ni-based cathode is trapped by their restrictive energy density, uncontrolled oxygen evolution reactions (OER) and sluggish reaction kinetics (Shen et al., 2021; Wang K. et al., 2023; Smith and Berlinguette, 2016). Compared to the high theoretical capacity of Zn anodes, Ni-based cathodes typically suffer from comparatively low capacity (like 290 mAh g^−1^ of β-Ni(OH)_2_) (Kimmel et al., 2021). Therefore, the practical energy density for assembled AZNBs is about 70 Wh kg^−1^, merely 20% of their theoretical level (Gong et al., 2014). Besides, Ni-based cathodes generally exhibit the oxygen evolution side reaction during the charging process, thereby inducing the low energy efficiency and serve capacity decaying of Zn-Ni batteries (Xie et al., 2023). More seriously, unoptimized Ni-based cathodes exhibit poor conductivity and high charge transfer impedance, consequently resulting in poor rate capacity of AZNBs (Zhang et al., 2022). Therefore, the development of high-performance cathode materials with optimized structures and components is essential for high-energy, fast-charging and long-lifespan Zn-Ni batteries. Very recently, metal-organic frameworks (MOFs) and covalent-organic frameworks (COFs) are proposed as the novel cathodes for high-capacity and high-rate alkaline Zn batteries (Li M. et al., 2018). For instance, Li et al. systematically introduced the latest advancements in Ni-based composite materials for supercapacitors electrodes (Li J. et al., 2024). Kang et al. developed a novel aqueous rechargeable Ni/Bi battery based on highly porous Bi_2_WO_6_ and Co_0.5_Ni_0.5_MoO_4_ microspheres as electrode active materials, which demonstrates a specific capacity of 179.2 mAh g^−1^ at 1 A g^−1^ and outstanding rate capability of 74.7% at 20 A g^−1^ (Kang et al., 2023). Notably, a few reviews about battery failure mechanisms, critical bottlenecks and mostly recent developments for Ni-based cathodes have been comprehensively delivered for high-performance AZNBs.

Herein, this review comprehensively focuses on the timely summary of representative Ni-based cathode materials and their corresponding energy storage chemistry in AZNBs. Subsequently, the key scientific challenges currently faced by alkaline Ni-based cathodes are introduced, specifically from the perspectives of limited energy density, inevitable side reactions, and the sluggish kinetics. Furthermore, design strategies including nanostructured optimization, defect engineering, ligand engineering, ion doping, and heterostructure regulation have been collected for high-performance Ni-based cathode materials. The final target of the ideal AZNBs should have high-performance electrode materials with low cost, durability and environmental suitability for the grid storage applications. Thus, prospective opportunities in the construction of more consummate Ni-based cathodes with novel reaction chemistry are brought forward to provide valuable directions, providing a fundamental guidance for future designing and development of advanced AZNBs.

## 2 Overview of Ni-based cathodes

**FIGURE 1:**
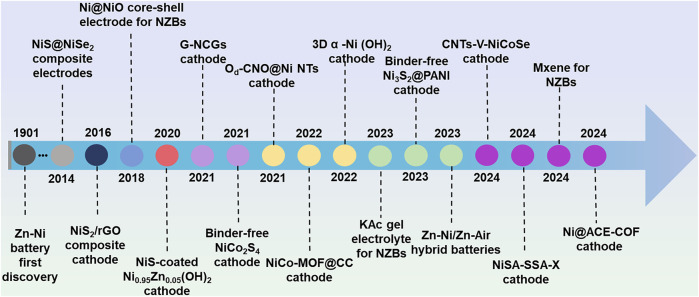
Main progress and brief development history of Ni-based cathode materials in aqueous Zn batteries in the past decade.

The development of AZNBs is closely related to the extensive exploration and establishment of various cathode materials, which are pivotal in determining the working voltage and theoretical capacity of the battery system (Huang M. et al., 2019). Specifically, Ni-based cathodes, in particular, contribute to an output voltage of approximately 1.75 V and the excellent low-temperature performance, retaining 84.1% of the capacity at −40°C (Chen S. et al., 2022). Over the past few years, a diverse range of Ni-based micro/nanostructures have been developed as cathode materials for AZNBs, demonstrating enhanced electrochemical performance and stability. Edison’s pioneering Zn-Ni battery emerged in 1901, and various Ni-based cathode materials like (Y(OH)^3−^coated Ni(OH)_2_, NF@NiO, GF-CNT@NiO) and their hybrids with carbon nanomaterials have been developed from 1901 to 2014 (N. Sac-Epee et al., 1997; Cheng et al., 2005; Liu et al.,2012). Up to now, lots of efforts have been made to realize Zn-Ni batteries with better performance and the development of novel Ni-based material exhibits a spiralrising feature. A summary of the major advancements and a brief development history of Ni-based cathode materials is presented in Figure 1.

### 2.1 Nickel hydroxide

**FIGURE 2:**
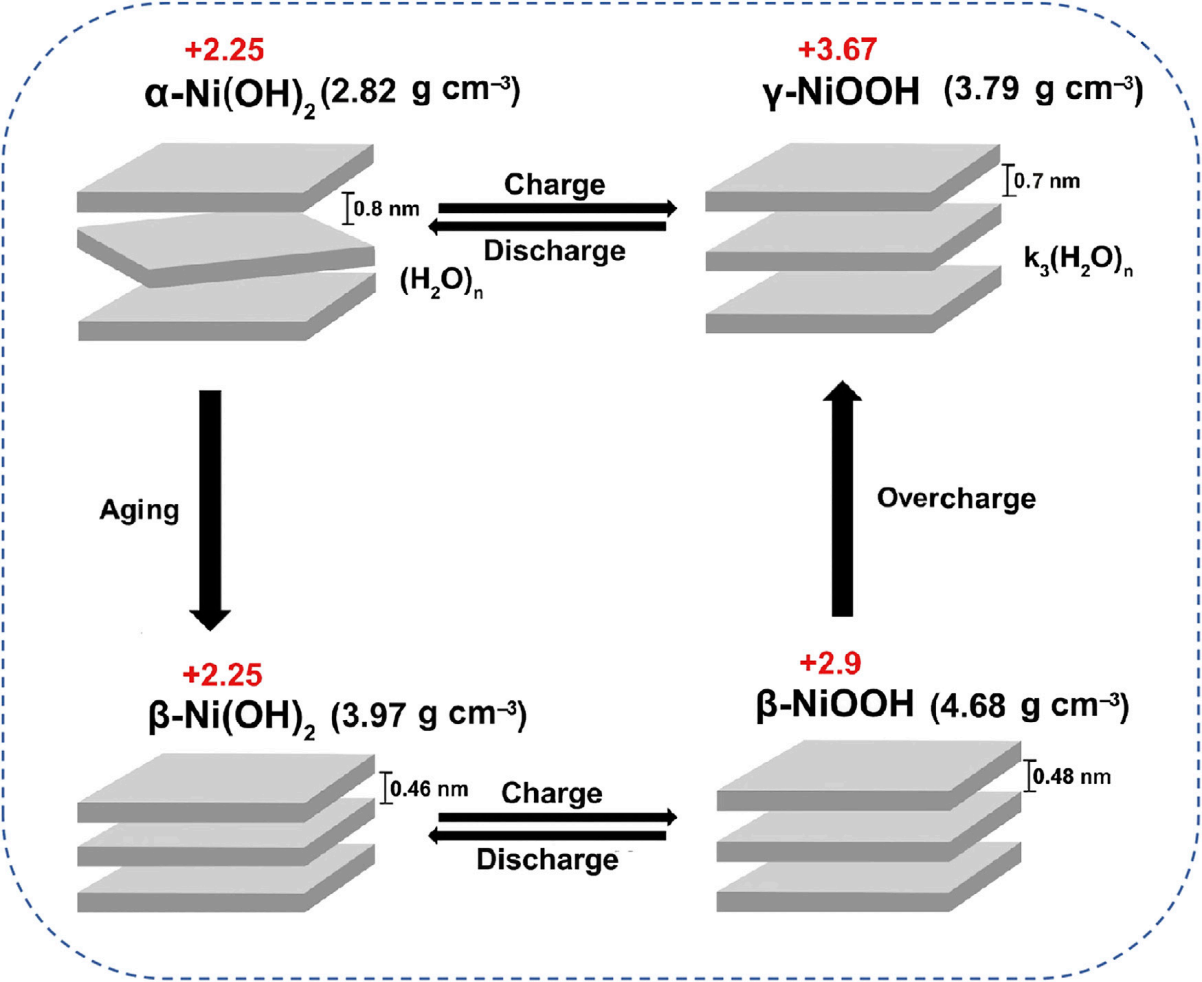
Bode cyclic graph of phase transition of Ni(OH)_2_/NiOOH reactions during charging and discharging processes in AZNBs. Reprinted with permission from Qin et al. (2021). Copyright (2021) John Wiley and Sons.

To date, commercial Ni(OH)_2_ as cathodes have been utilized to power high-power and high-security equipment, demonstrating effective supplements for LIBs (Hao et al., 2019). To be specific, Ni(OH)_2_ is particularly distinguished within the family of Ni-based cathodes due to its high specific capacity and good reproducibility. Notably, Ni(OH)_2_ exists in two main crystal forms of α-Ni(OH)_2_ and β-Ni(OH)_2_ (Shruthi et al., 2016; Zhang et al., 2015). Thereinto, β-Ni(OH)_2_ cathode characterized by good crystallinity generally undergoes two distinct reaction stages of “ ${\text{Ni}\left(\text{OH}\right)}_{2}+{\text{OH}}^{-}-{\;e}^{-}⇌\text{ NiOOH}$ ” and “ ${4\text{OH}}^{-}-{\;4\;e}^{-}⇌O_{2}+2H_{2}O$ ” during charging process. Notably, the second stage involves a side reaction known as the oxygen evolution reaction (OER) in which should be suppressed as much as possible to prevent loss of energy and degradation of Ni-based cathode materials. Furthermore, Ni(OH)_2_ cathodes experience complex phase transitions during the charge/discharge process. As described by the Bode mechanism in Figure 2, β-Ni(OH)_2_ phase can only convert into β-NiOOH possessed poor crystallinity.” Yet, the β-Ni(OH)_2_/β-NiOOH transformation involved only 1 M transferred electrons per formula unit transfer limits the theoretical capacity of β-Ni(OH)_2_ cathode (290 mAh g^−1^). In contrast, α-Ni(OH)_2_ cathodes can reversibly convert into γ-NiOOH involving the transfer of 1.67 M of electrons per formula unit, resulting in a higher theoretical capacity of 480 mA h g^−1^ for AZNBs (Qin et al., 2021).

In addition, α-Ni(OH)_2_ features a larger interlayer spacing than that of β-Ni(OH)_2_, which facilitates proton and electron migration for boosted reaction kinetics and rate capacity of Zn-Ni batteries. Moreover, this interlayer structural endows α-Ni(OH)_2_ with less volume changes and better cycling stability compared to β-Ni(OH)_2_ (Zhang et al., 2014). However, α-Ni(OH)_2_ cathode is prone to aging into β-Ni(OH)_2_ due to its instability in alkaline solution, and β-Ni(OH)_2_ can further irreversibly transform into γ-NiOOH during charging, thereby leading to a decline in battery performances (Ash et al., 2006). More seriously, the phase transitions within Ni(OH)_2_ cathodes are also associated with volume expansion and contraction, resulting in the deterioration of capacity, rate performance, and cycle stability of Zn-Ni batteries (Liu et al., 2020). Furthermore, NiOOH generally acts as an effective oxygen evolution reaction (OER) catalyst (Diaz-Morales et al., 2016). Therefore, the inevitable oxygen evolution side reaction at the cathode side poses significant challenges for achieving high energy efficiency and long-lifespan in AZNBs.

### 2.2 Nickel sulfides

**FIGURE 3:**
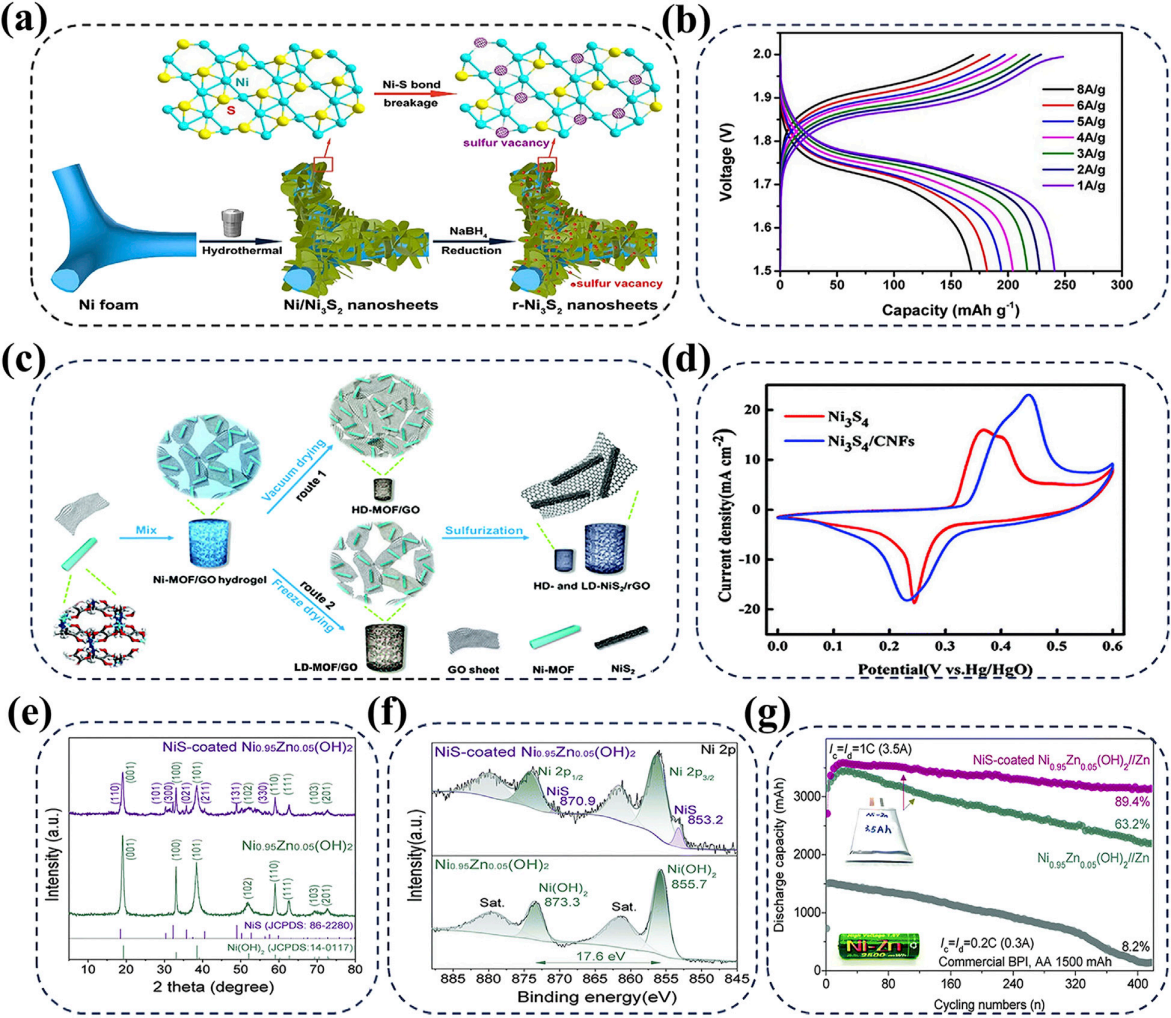
**(A, B)** Schematic illustration for the fabrication of the r-Ni_3_S_2_ NS electrode with sulfur vacancies and rate performance of r-Ni_3_S_2_//Zn battery. Reprinted with permission from He et al. (2020). Copyright (2020) American Chemical Society. **(C)** Preparation process of NiS_2_/rGO for high performance AZNBs. Reprinted with permission from Shi et al. (2019). Copyright (2019) Royal Society of Chemistry. **(D)** The CV profiles of Ni_3_S_4_ and Ni_3_S_4_/CNFs at 20 mV s^-1^. Reprinted with permission from Bao et al. (2021). Copyright (2021) Royal Society of Chemistry. **(E–G)** XRD and XPS Ni 2p spectra of Ni_0.95_Zn_0.05_(OH)_2_, NiS-coated Ni_0.95_Zn_0.05_(OH)_2_, respectively and cycling performance of 3 Ah Zn//NiS-coated Ni_0.95_Zn_0.05_(OH)_2_ pouch cell in comparison with the commercial BPI Ni-Zn battery. Reprinted with permission from Zhou et al. (2020c). Copyright (2020) Royal Society of Chemistry.

Due to their higher electronic conductivity and superior structural stability compared to Ni-based oxides and hydroxides, nickel sulfides including NiS_2_, Ni_3_S_2_, and Ni_3_S_4_ exhibit remarkable performance in the field of optoelectronics (Wei et al., 2019; Mi et al., 2015; Wei et al., 2014). As for AZNBs, Ni_z_S_x_ cathodes generally experience the redox reaction of “ ${\text{Ni}}_{z}S_{x\;}+y\;{\text{OH}}^{-}⇌{\text{Ni}}_{z}S_{x}{\left(\text{OH}\right)}_{y}+y\;e^{-}$ ” (Gao et al., 2018). Notably, the multiple valence states of nickel allow it to form a variety of compounds with sulfur elements, thereby resulting in distinct electrochemical properties of nickel sulfide cathodes (Dai et al., 2013). Moreover, the low electronegativity of sulfur ions enables nickel sulfide cathodes with relatively stable structures, which effectively protect and facilitate electron transfer for high-rate and robust Zn-Ni batteries (Krishnamoorthy et al., 2014). Among the nickel sulfides, Ni_3_S_2_ shows significant potential for AZNBs due to its high electronic conductivity (5.5 × 10^4^ S cm^−1^ at room temperature) and highly reversible redox reactions in alkaline solution (Guan et al., 2015; Ke et al., 2015). For instance, He et al. developed a novel binder-free r-Ni_3_S_2_ nanosheets supported on Ni foam as the cathode for AZNBs, which demonstrated an impressive specific capacity of 240.8 mAh g^−1^ at 1 A g^−1^ and remarkable rate capability (Figures 3A,B) (He et al., 2020). However, the high capacity and cyclic durability of the Ni_3_S_2_ cathode still need improvement for high-performance Zn-Ni batteries. Due to the fast diffusion of electrolyte ions, high electrical conductivity, and abundant active sites, NiS_2_/reduced graphene oxide (NiS_2_/rGO) composite cathode exhibits enhanced stability and energy density of 357.7 Wh kg^−1^ for advanced AZNBs (Figure 3C) (Shi et al., 2019).

Despite these significant advancements, the design of transition nickel sulfide cathodes is still limited by insufficient reaction activity and kinetics (He et al., 2017). Recently, Bao et al. proposed Ni_3_S_4_/CNFs cathode which uses carbon nanofibers (CNFs) as support materials for the growth of Ni_3_S_4_ nanoparticles (Bao et al., 2021). The addition of conductive CNFs not only exposes more active sites, but also accelerates electron transfer to improve the battery performance. Furthermore, due to the binding effect of oxidized CNFs with oxygen functional groups, Ni_3_S_4_/CNFs hybrid cathode exhibits a larger redox peak intensity compared to bare Ni_3_S_4_ at 20 mV s^−1^, which suggests the improved capacity of 215 mAh g^−1^ in Ni_3_S_4_/CNFs cathode (Figure 3D). In addition, the similar significant results have been achieved in α-Ni(OH)_2_ cathode wrapped by NiS protective layer (Figures 3E,F) (Zhou et al., 2020c). Zhou et al. constructed ultra-dense NiS-coated Co-free Ni_0.95_Zn_0.05_(OH)_2_ cathode based on the anion exchange reaction and Kirkendall effect, which combines the rich ion transmission pathways of α-Ni(OH)_2_ with the excellent conductivity of NiS. The obtained 3-Ah Zn-Ni pouch cell exhibits an exceptionally high energy density of 506 Wh L^−1^ and outstanding long-term durability over 400 cycles, as shown in Figure 3G. Therefore, rational designing nickel sulfide cathodes with optimized surface and reaction activity is essential for the fast-charging and high-energy AZNBs with long lifespan.

### 2.3 Nickel oxide

Various NiO-based nanoarchitectures, such as NiO nanoflakes, NiO nanosheets, and Co_3_O_4_@NiO arrays, have been extensively explored as cathodes for AZNBs, demonstrating notable electrochemical performance and stability (Liu et al., 2016; Li et al., 2020). The excellent two- or three-dimensional ordered ion transport channels formed within NiO cathode enable rapid ion migration during electrochemical reactions, making NiO-based materials strong candidates for advanced AZNBs (Zeng et al., 2018). Despite the same electron transfer number as Ni(OH)_2_, NiO cathode exhibits a higher theoretical capacity (360 mAh g^−1^) and better cycling stability due to its lower molecular weight and simpler composition (Yin et al., 2022). However, the inherently large size of NiO leads to extended ion/electron transfer paths during electrochemical processes, which slows down rate performance of Zn//NiO batteries during charge/discharge cycles (Wang et al., 2007). Current research on transition metal oxides (TMOs) primarily focuses on developing multiple valence hosts, reducing particle size, increasing surface area to shorten ion/electron transfer paths, improving oxide conductivity, and using doping modifications to enhance cycle stability by forming more stable oxides (Wang et al., 2018).

**FIGURE 4:**
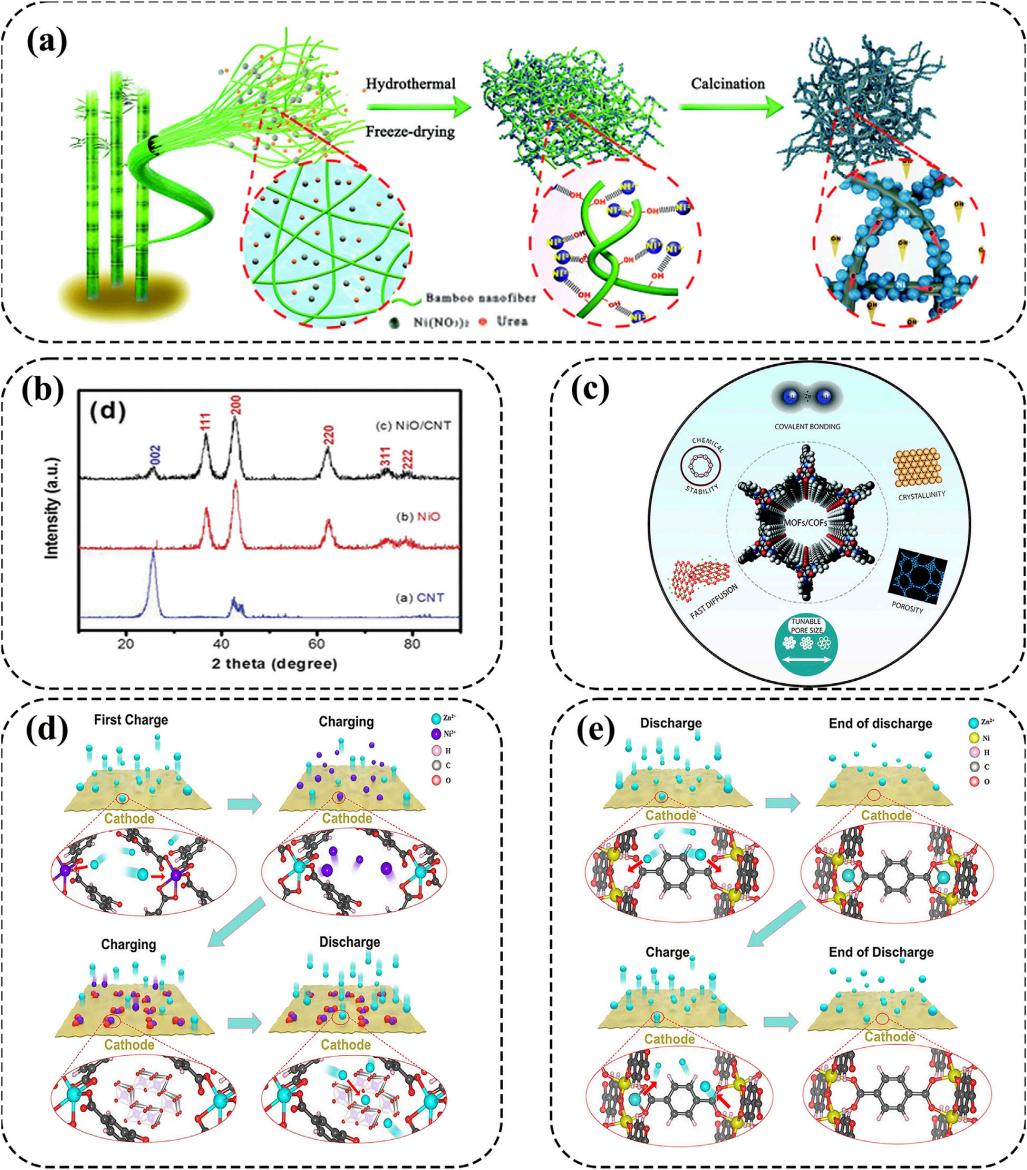
**(A)** The schematic diagram to prepare Ni-BCF materials. Reprinted with permission from Jiang et al. (2020). Copyright (2020) Royal Society of Chemistry. **(B)** X-ray diffraction patterns of CNT, NiO and NiO–CNTs. Reprinted with permission from Wang et al. (2015). Copyright (2015) Royal Society of Chemistry. **(C)** The most important properties of MOFs and COFs. Reprinted with permission from Zhao X. et al. (2021). Copyright (2021) Royal Society of Chemistry. **(D, E)** Schematic diagram of metal ion dissolution/deposition and Zn^2+^ insertion/extraction mechanisms of pristine MOF cathodes. Reprinted with permission from Zhang D. et al. (2024). Copyright (2024) Elsevier.

Recently, Jiang et al. developed robust Ni-NiO nanoparticles as cathodes by configuring nanostructured Ni and Ni/NiO nanoparticles onto bamboo-derived cellulose nanofibers (BCF) (Jiang et al., 2020). The unique hierarchical 3D network of Ni-NiO cathode allows for easy electrolyte penetration and rapid ion/electron transport, contributing to high energy density (313.4 Wh kg^−1^) and good cycling stability over 1000 cycles (Figure 4A). Besides the substrate BCF, Wang et al. prepared NiO nanoflakes as cathode materials onto the supported carbon nanofibers (Figure 4B) (Wang et al., 2015). Attributed to the ultra-thin nanosheets, shorter electron transmission distance, and higher crystallinity, NiO-CNT cathode exhibits improved electrical conductivity to enhance the specific capacity and energy density for high-performance AZNBs. Thus, NiO-based cathodes with nanoparticles and modified element components is beneficial for the high conductivity and redox active sites for high-performance Zn-Ni batteries.

### 2.4 Metal-organic and covalent-organic frameworks

Metal-organic frameworks (MOFs) and covalent-organic frameworks (COFs) possess unique features, including high porosity, tunable pore size, large specific surface areas and customizable chemistry (Figure 4C) (Zhao et al., 2022; Chen W. et al., 2024; Peng et al., 2023; Zhao X. et al., 2021). Thus, MOFs and COFs in electrochemical energy storage have distinct advantages, especially in AZNBs. First and foremost, the porous nanostructures with high porosity and large specific surface areas are beneficial to high charge storage capacity of Zn-Ni devices; simultaneously, the tunability of pore sizes make it possible to optimize the ion transport channels, thereby potentially improving the ion diffusion and reaction kinetics of these MOF and COF cathodes (Zhang D. et al., 2024). Owing to these attributes, MOFs and COFs hold powerful competitive advantages over other traditional materials and tremendous promise in AZNBs (Peng et al., 2023; Xing et al., 2024). For instance, Chen et al. reported that NiCo-MOF@CC-3 (NCM@CC-3) cathode with an optimized Co/Ni ratio of 1:1 showed excellent electrical conductivity based on density functional theory (DFT) calculations (Chen T. et al., 2022). As a result, the NCM@CC-3//Zn@CC battery featured a high areal energy density of 2.97 mWh cm^−2^, and superior capacity retention of 83% after 6000 cycles. Besides, Chen et al. introduced a novel C_3_-symmetrical azacrown ether (ACE) building block, tris(pyrido)[18]crown-6 (TPy18C6), for the preparation of ACE-COFs with strengthened Ni^2+^ immobilization through reticular synthesis (Chen Q. et al., 2024). As a result, Ni@ACE-COF cathode displayed ultra-high areal capacity (∼1.27 mAh cm^−2^ at 8 mA cm^−2^) and excellent cycle stability (>1000 cycles) for superb AZNBs.

Currently, the energy storage mechanisms of MOFs and COFs are lacking systematic summary to boost their further development. For MOFs, there are two main reaction chemistry of metal ion dissolution/deposition and Zn^2+^ insertion/extraction mechanism, as shown in Figures 4D,E. As for the metal ion dissolution/deposition mechanism, metal ions are dissolved into the electrolyte to become electrochemically active materials and subsequently deposited on the cathode surface during the battery working. And in Zn^2+^ insertion/extraction mechanism, Zn^2+^ inserts into the MOF structure and binds with the active sites within the framework, promoting energy exchange and charge transfer for high-performance Zn//MOF batteries (Zhang D. et al., 2024). Previous studies show that n-type COF cathodes, which acquire electrons and reduced to anions, exhibit pure Zn^2+^ intercalation or Zn^2+^/H^+^ co-storage mechanisms for AZNBs (Lu et al., 2024). Thus, regulating the active sites and reaction mechanisms of MOF and COF cathodes can effectively broaden the research scope of Zn-Ni batteries.

### 2.5 Layered double hydroxide

Layered double hydroxides (LDH), commonly known as hydrotalcite-like compounds, represent a unique class of layered materials composed of positively charged layers intercalated with anions, to maintain charge equilibrium (Hu et al., 2021). The intermediate anions and layers of Ni-based LDHs are loosely bound and can often be exchanged. Among the reported nickel-based cathode materials, LDHs are regarded as promising candidates due to their high capacity, long cycle life, and tunable properties, making them extensively studied and utilized as cathodes in Ni-Zn batteries. The general formula for LDHs is [M(II)_1-x_M(III)_x_ (OH)_2_] ^x+^(A^n−^)_x/n_mH_2_O, where M(II) is a divalent metal cation, M(III) is trivalent metal, and A^n−^ is an anion. The common LDH used in the cathode material of nickel-zinc batteries is: Ni-based LDHs and Mixed LDHs (Chen et al., 2019). The most common type among LDHs is nickel cobalt layered double hydroxide, designed as NiCo-LDHs (Chen et al., 2019; Jiang et al., 2023). Specifically, NiCo-LDH cathode materials combine the advantages of nickel and cobalt with good conductivity and redox reversibility, contributing to the high capacity and good cycling stability of AZNBs.

**FIGURE 5:**
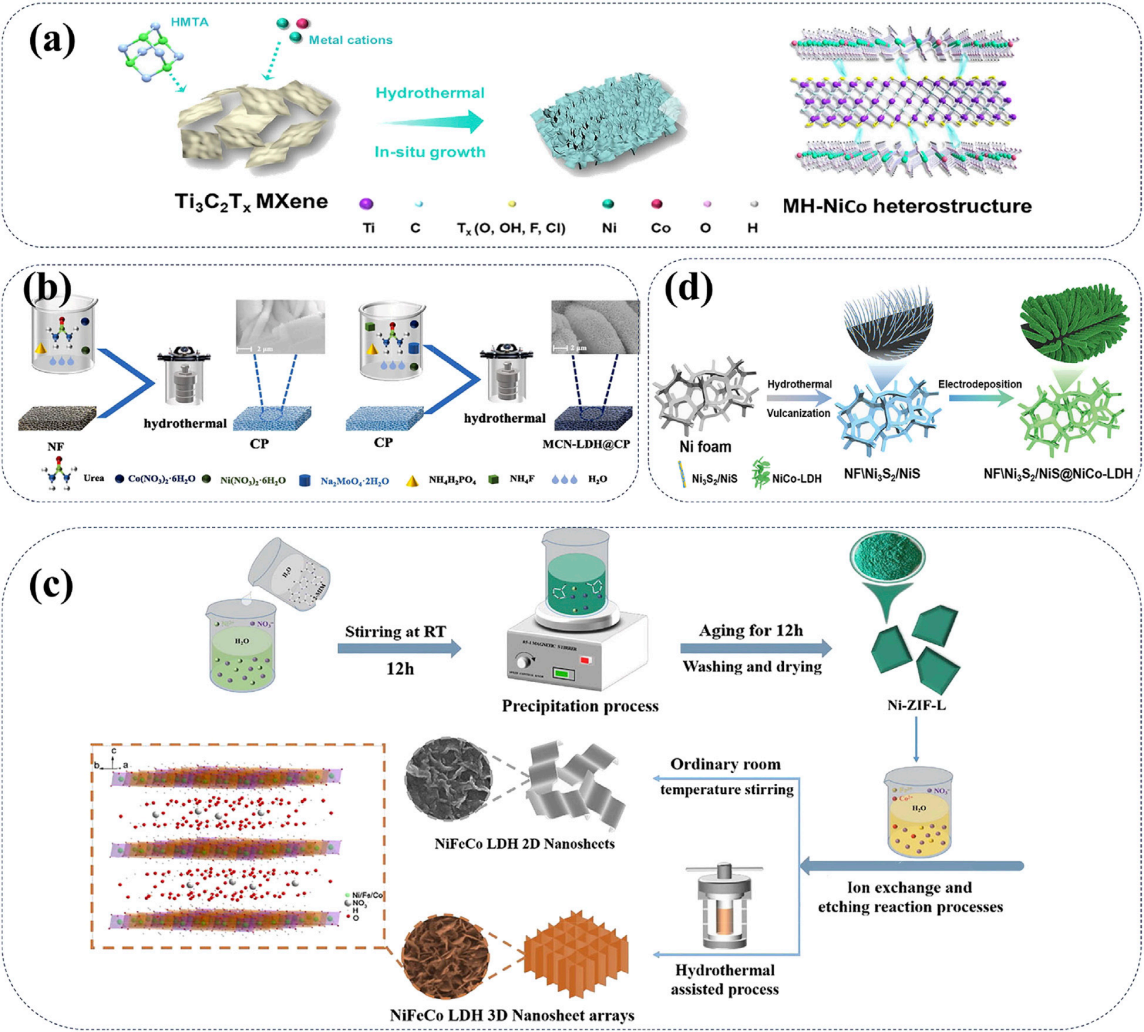
**(A)** Schematic synthesis process of MH-NiCo heterostructure, 2D-2D Heterostructured Nanomaterials. Reprinted with permission from Zhang et al. (2024c). Copyright (2024) John Wiley and Sons. **(B)** Schematic preparation illustration of MCN-LDH@CP with bilayered structure. Reprinted with permission from Ma et al. (2024). Copyright (2024) Elsevier. **(C)** Schematic illustration of the synthesis process of NiFeCo LDH nanoplate arrays with 3D porous structure. Reprinted with permission from Wang H. et al. (2023). Copyright (2023) Elsevier. **(D)** Schematic diagram of the synthesis of NF\Ni_3_S/NiS@NiCo-LDH with hierarchical heterostructure. Reprinted with permission from Zhou K. et al. (2022). Copyright (2022) John Wiley and Sons.

In recent years, researchers have prepared NiCo-LDH materials through different synthesis methods, such as hydrothermal method and co-precipitation method (Lu et al., 2020). And these NiCo-LDH cathode materials have been continuously modified to enhance their battery performances. Recently, two-dimensional (2D) MXene heterostructures are successfully synthetized to solve the common issues of inevitable aggregation and self-degradation (Chen Z. et al., 2024; Zhang G. et al., 2024). Due to the unique surface characteristics of MXenes, various NiCo-LDH nanomaterials can be induced onto the MXene substrates. This strategy effectively mitigates self-stacking defects and increases the exposure of surface area of the NiCo-LDH cathodes. In particular, 2D-MXene@NiCo-LDH (MH-NiCo) heterostructures exhibit enhanced structural stability, improved chemical reversibility, and higher charge transfer efficiency compared to pure NiCo-LDH (Figure 5A) (Zhang G. et al., 2024). Besides the modification of 2D-MXenes, zinc-induced phase reconstitution method can greatly improve the electrochemical stability of CoNi-LDH cathode materials (Pang et al., 2022). Specifically, the molybdate intercalation significantly increases the specific surface area as well as expands the interlayer spacing of CoNi-LDH cathodes, thus improving the ion diffusion kinetics and specific capacity of AZNBs. A typical molybdate intercalated cathode material of MCN-LDH@CP have been prepared via simple hydrothermal process for high capacity (1.74 mAh cm^−2^) and good cycle stability (97.8% after 7000 cycles) of Zn-Ni batteries (Figure 5B) (Ma et al., 2024).

Furthermore, mixed LDHs incorporating multiple metal ions are also frequently used as cathode materials for high-performance AZNBs. For example, NiFeCo-LDH, which combines nickel, cobalt, and iron, provides greater stability and higher energy density compared to single-metal LDHs (Wang H. et al., 2023). The synthesis process of NiFeCo-LDH nanoplate arrays is showed in Figure 5C. By controlling synthesis conditions, including the ratio of metal ions and the type of interlayer anions, the structure and properties of NiFeCo-LDH can be precisely tailored to meet the needs of advanced Ni-Zn batteries. Another mixed LDH cathode of NF\Ni_3_S_2_/NiS@NiCo-LDH has been fabricated with the self-supported 3D hierarchical heterostructures (Zhou K. et al., 2022). Notably, NF\Ni_3_S_2_/NiS@NiCo-LDH cathode with strong interfacial bonds provide abundant reactive sites, rapid ion diffusion channels, as well as strong structural stability for high capacity (434.5 mAh g^−1^) and long lifespan (over 5000 cycles) of Zn-Ni batteries. A schematic diagram of the synthesis of NF\Ni_3_S_2_/NiS@NiCo-LDH is shown in Figure 5D. Therefore, compositions and interlayer spacing of these Ni-based LDHs can be rational designed to optimize Ni-Zn battery performances.

### 2.6 Other cathode materials

In addition to the above five cathode materials, there are some other Ni-based cathode materials such as nickel molybdate (NiMoO_4_), nickel selenides (NiSe_2_) and nickel/cobalt-based materials (Zhou L. et al., 2020; Shen et al., 2020; Liu et al., 2023; Zhou L. et al., 2022). Specifically, NiMoO_4_ with superior conductivity is a typical low-cost and environmentally friendly binary nickel oxide cathode for AZNBs. As a result, NiMoO_4_-NC cathode exhibits a improved capacity of 229.2 mAh g^−1^ (3.4 A g^−1^) and cycling stability of 85.9% capacity retention after 3000 cycles (Zhou L. et al., 2020). Moreover, intrinsically Pauli-paramagnetic NiSe_2_ cathode has low resistivity (10^–3^ Ω cm) for high-rate and fast charging AZNBs (Zhou et al., 2020b; Wang et al., 2017). Recently, Zhou et al. proposed layered NiSe_2_ nanosheet arrays as a robust cathode for high-performance AZNBs, which showed excellent rate capability (58% capacity reservation at 72.8 A g^−1^), ultrahigh power density (91.22 kW kg^−1^) and extremely long lifespan (91.7% capacity retention after 10,000 cycles) (Zhou et al., 2020b).

Nickel/cobalt-based cathode materials have the same advantages as NiMoO_4_, except for poor electrical conductivity and insufficient electrochemically active sites. To address these issues, various strategies including defect engineering, structural design, element doping and composition regulation, have been reported to optimize the capacity and cycling stability of nickel/cobalt-based cathode materials (Liu et al., 2023). For instance, Liu et al. synthesized S-NCO cathode by hydrothermally doping sulfur into NiCo_2_O_4_ to improve the lifespan without capacity fading after 4000 cycles (Liu et al., 2023). Moreover, Zhou et al. prepared bimetallic cobalt-nickel phosphate octahydrate (NCP) with hierarchical structure *in situ* on nickel foam by one-step hydrothermal method (Zhou L. et al., 2022). Regulating the composition of cobalt and nickel elements, Zn//NCP battery exhibits a high areal energy density of 5.42 mWh cm^−2^ and a maximum power density of 129.68 mW cm^-2^. Thus, exploring novel Ni-based cathodes with new redox reactions is of importance for advanced AZNBs.

## 3 Challenges for Ni-based cathodes

Despite the significant success achieved since the commercialization of Ni(OH)_2_, optimization of cathode materials still face several challenges for advanced AZNBs. Early research primarily focused on the development of new materials and morphology control in electrodes and electrolyte. However, inadequate energy density, inevitable side reaction, and slow dynamics become visible issues with the deepening of studies. Thus, these challenges pose significant obstacles to the commercialization of AZNBs and must be thoroughly addressed for the technology to reach its full potential.

### 3.1 Inadequate energy density

The energy density of a battery, which refers to its ability to store energy, is crucial for meeting the endurance requirements of electronic devices. It is closely related to both the discharge capacity and operating voltage of the battery. Notably, AZNBs are distinguished within the AZIBs family by their high discharge voltage platform (∼1.75 V), which surpasses that of ordinary aqueous Zn batteries with mild/acid electrolytes (typically <1.5 V) (Li W. et al., 2018). However, the energy density of assembled AZNBs falls short of expectations due to the relatively lower capacity of Ni-based cathodes compared to zinc anodes and their lower utilization rates (Fang et al., 2018). To overcome this barrier, significant efforts have been made to develop cathode materials with higher specific capacities, as discussed earlier. Nevertheless, most of these materials rely on a reversible redox process involving a single electron transfer, which inherently limits their theoretical specific capacity (Liu et al., 2019). Furthermore, the redox potential of electrode materials is intrinsically tied to their crystal structure and configuration, making it difficult to alter (Lu et al., 2012). Thus, expanding the operating voltage seems to be an attractive option to improve energy density.

**FIGURE 6:**
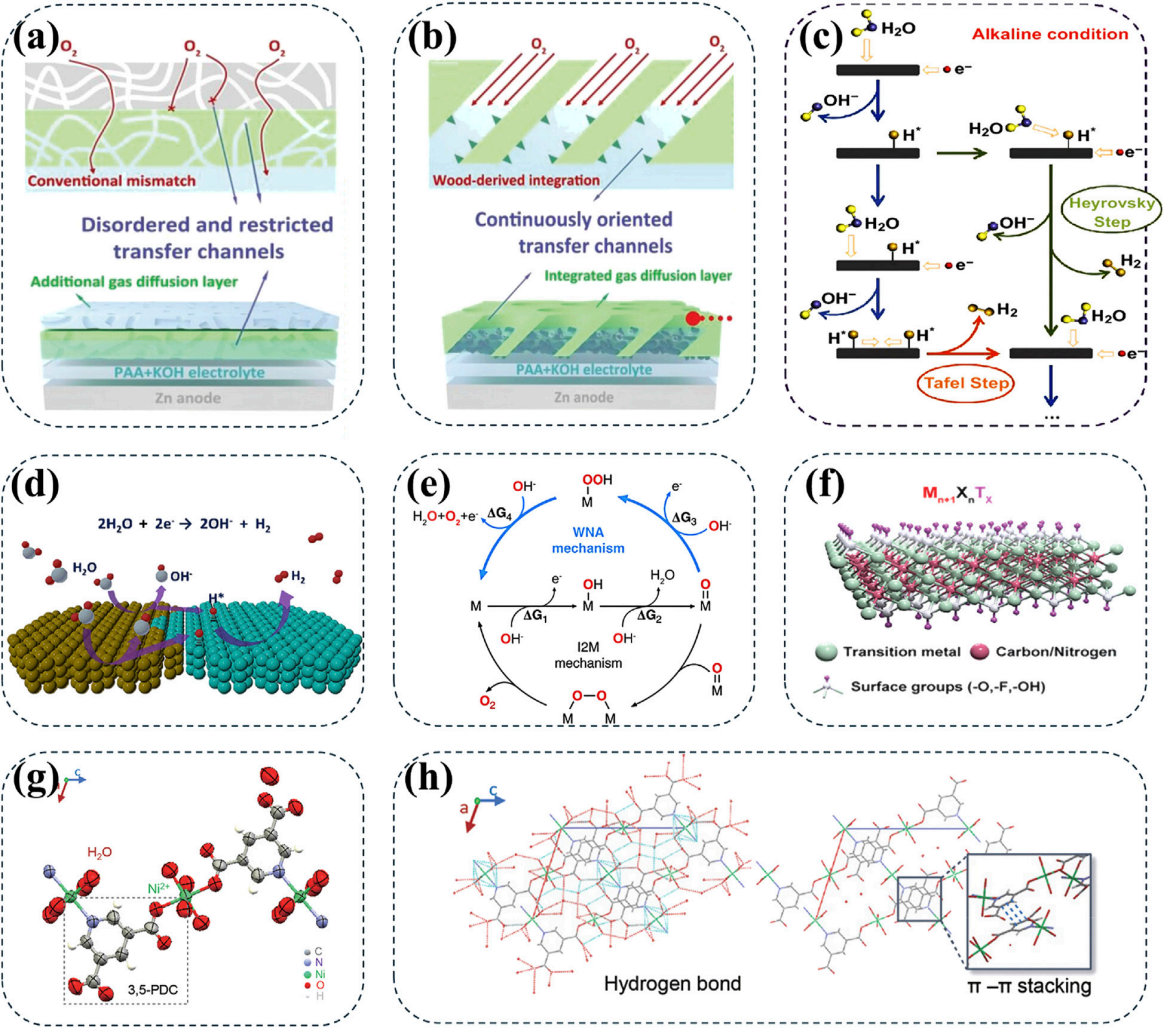
Comparison of **(A)** conventional three-phase interfacial channels with imperfect matching and disordered transfer feature and **(B)** continuously oriented three-phase interfacial channels with 1D directional transfer characteristics in hybrid AZNBs. Reprinted from Li L. et al. (2023). Copyright (2023) John Wiley and Sons. **(C, D)** Schematic pathways and the process of H_2_ evolution in alkaline medium. Reprinted with permission from Wang H. et al. (2024). Copyright (2024) Elsevier. **(E)** OER cycling process of two different mechanisms. Reprinted with permission from Zhao et al. (2023). Copyright (2023) American Chemical Society. **(F)** Ni-based MXene hosts for high conductivity and reaction catalysis. Reprinted with permission from Wang Z. et al. (2024). Copyright (2024) John Wiley and Sons. **(G, H)** NCGs constructed by the coordination of Ni^2+^ with organic ligands for improved redox kinetics and 3D stacks formed by hydrogen bonds and π-π stacking interactions to provide rapid electron/ion transport channels. Reprinted with permission from Zhang et al. (2021). Copyright (2021) John Wiley and Sons.

From the perspective of battery design, another strategy of designing a Zn-based hybrid batteries has been used to broaden the operating voltages (Li et al., 2017). Due to similar electrolyte systems and well-matched discharge voltage platforms of Zn-Ni and Zn-Air batteries, their electrochemical reactions can be technically integrated within the hybrid devices (Huang Z. et al., 2019). Specifically, the assembled hybrid battery can achieve a high energy density of 644.7 Wh kg^−1^ and good durability in a wide operating voltage range of 0.6–2.0 V (Li L. et al., 2023). More importantly, compared to standalone AZNBs, the hybrid Zn batteries with continuously oriented three-phase reaction interface channels can significantly improve the kinetics of electrocatalytic oxygen reaction with a specific capacity by about 370%, making them one of the most excellent alkaline Zn batteries (Figures 6A,B). Inspired by this, alkaline zinc/lithium hybrid batteries have been designed to provide an unprecedented operating voltage of 3.41 V and a high energy density of 362.4 Wh kg^−1^, approaching the energy density of commercial LIBs (Chen et al., 2023).

Compared to the strategy of developing high-capacity cathode materials to boost the energy density, designing multiple electrochemical reactions is considered as a more effective potential strategy for high-voltage AZNBs (Cui M. et al., 2021). Although optimized electrolytes can effectively widen the battery working window, they often fail to achieve satisfactory energy density due to mismatches with hybrid batteries (Manna et al., 2022). Therefore, achieving a well-matched reaction interface is crucial for the success of hybrid power batteries. Furthermore, achieving both ultra-high volumetric and gravimetric energy density remains a challenge in reported AZNBs (Chen Z. et al., 2024). Hence, the continuous search for more cathode materials with theoretical capacity higher than that of Zn metal (820 mAh g^−1^) would be a significant advancement in the reaction designed hybrid Zn batteries.

### 3.2 The inevitable side reactions

The inevitable HER and OER along with the release of H_2_ and O_2_ at the anode and cathode sides, respectively, present severe issues for low energy efficiency, degradation of cathode materials, and zinc dendrite growth (Xiao et al., 2022; Yang et al., 2024; Xie et al., 2023; Li et al., 2021). Moreover, the side reaction also causes swelling of the AZNBs in a closed system, leading to short circuits or battery expansion in practical applications. Specifically, the redox couple of Zn/Zn^2+^ presents a standard electrode potential (−0.76 V vs. SHE), which means the hydrogen evolution reaction is thermodynamically preferred at the anode/electrolyte interface (AEI), thus leading to the production of H_2_ (Wang H. et al., 2024; Yang H. et al., 2021; Dundálek et al., 2017). In aqueous electrolytes, the HER is a two-electron transfer reaction and the specific synthesis of a hydrogen molecule through either Volmer-Heyrovsky or Volmer-Tafel mechanism (Figure 6C). Different to the mild or acid Zn batteries, the HER pathways and total reaction equation can be concluded as $2H_{2}O+2e^{-}\to \;2{\text{OH}}^{-}+H_{2}$ under alkaline conditions (Figure 6D). It is well-established that H_2_ catalytic activity and exchange currents are significantly lower in an alkaline medium than that in acidic solutions (Wei J. et al., 2018). Yet, the strategies for the inhibition of HER still need to develop for high efficiency and robust Zn-Ni batteries, especially for the large-scale energy storage systems.

Compared to HER, OER at the cathode side deserves more attention in AZNBs during the charging process. Based on previous studies, the overall OER can be concluded by the equation of $4{\text{OH}}^{-}\to O_{2}+2H_{2}O+4e^{-}$ . And the OER occurs by two different ways: water nucleophilic attack (WNA) or the adsorbate evolution mechanism (AEM), and the interaction of intermolecular O-O coupling (I2M). Specifically, the WNA process and the paths of I2M have been depicted in detail for the generation O_2_ during battery charging, as depicted in Figure 6E (Zhao et al., 2023). Notably, all these reactions are assumed to proceed through proton-coupled electron (or hole) transfers (PCET) (Yang X. et al., 2021). Once OER happens, the Ni-based cathodes will undergo low reaction efficiency and inferior phase reversibility for high-energy and long-lifespan AZNBs. For example, OER as a competitive reaction of the conversion between Ni_3_S_2_ and Ni_3_S_2_(OH)_x_ during Ni_3_S_2_ cathode charging results in energy loss and instability of the cathode materials (Hu et al., 2015). In previous studies, Joseph F. Parker and co-workers used combination of additives and controlled the charging voltage to suppress OER, but actually these methods are insufficient to solve the OER issue completely, and will increase the cost of electrolytes as well as cause capacity loss (Parker et al., 2017). Therefore, understanding mechanisms of OER and HER is important for the design of high-performance Ni cathodes and durable Zn anodes.

### 3.3 The sluggish kinetics

Sluggish kinetics caused by the poor conductivity, slow ion diffusion rate, and high charge transfer impedance is crucial for advancing practical applications of AZNBs. Thus, current researches are focused on overcoming this challenge to enhance the overall performance of AZNBs (Wang K. et al., 2023; Chen X. et al., 2024; Tian et al., 2022). Specifically, Ni-based oxide cathodes typically suffer from low capacity and poor stability, largely due to their slow electron/ion transport rates and poor electrochemical reversibility during the phase transition process. For example, Ni(OH)_2_ in the cathode undergoes reversible conversion to NiOOH to release the stored energy during discharging. Then, Ni(OH)_2_ is reversibly oxidized to form NiOOH, storing electrical energy in the charging process. Therefore, the phase transition kinetics directly limits the energy conversion efficiency and rate capability of AZNBs (Qin et al., 2021). Moreover, the phase transformation process can lead to structural changes in the Ni-based cathode materials, affecting the ion diffusion kinetics and potentially reducing the overall battery performance. Besides, Ni-based cathodes exhibit lower ionic conductivity, aggravating sluggish ion diffusion kinetics and poor rate performance of AZNBs. Recently, various strategies including cathode modification, electrolyte optimization and battery design have been employed to enhance the conductivity, reduce charge transfer resistance, and minimize structural degradation, respectively, for fast-charging AZNBs (Tian et al., 2022; Huang M. et al., 2019). Yet, the inherent poor conductivity and significant crystal structure collapse of Ni-based cathodes still need further investigate from the fundamental of reaction mechanisms.

The lack of sufficient active sites and electron and ion transport channels for the reaction results in the sluggish kinetics. Finding novel materials for Zn-Ni battery cathodes to provide abundant active sites and fast electron ion transport channels is a major challenge. For example, based on the continuous electron transport networks, the functional groups (such as -OH) on the surface of MXene can enhance the adsorption and catalysis of nickel-based materials, thereby improving the overall performance of AZNBs (Figure 6F) (Wang Z. et al., 2024). Additionally, constructed by the coordination of nickel ions with organic ligands (Figure 6G), the Ni (II) coordination supramolecular grids (NCGs) forms 3D stacks through hydrogen bonds and π-π stacking interactions to provides rapid electron and ion transport channels for high-rate Zn-Ni batteries (Figure 6H) (Zhang et al., 2021). While searching for these new materials, it is also necessary to consider the possibility of reactions between the electrode material and the alkaline electrolyte, which can deplete the active material and reduce the efficiency of the battery. Therefore, sluggish kinetics of Ni-based cathode materials needs to be solved through innovative research and continuous development of new materials for high-performance AZNBs.

## 4 Design strategies for Ni-based cathodes

To circumvent the above challenges, great research efforts have been dedicated to the optimization of Ni-based cathodes. Here, we summarize and highlight several feasible electrode design strategies including defect engineering, ligand engineering, ion doping, heterostructure regulation, nanostructured optimization. Furthermore, a single optimization strategy is not sufficient to solve all the problems of nickel cathodes. Therefore, it is recommended to adopt multiple strategies simultaneously to achieve high-performance AZNBs.

### 4.1 Defect engineering

**FIGURE 7:**
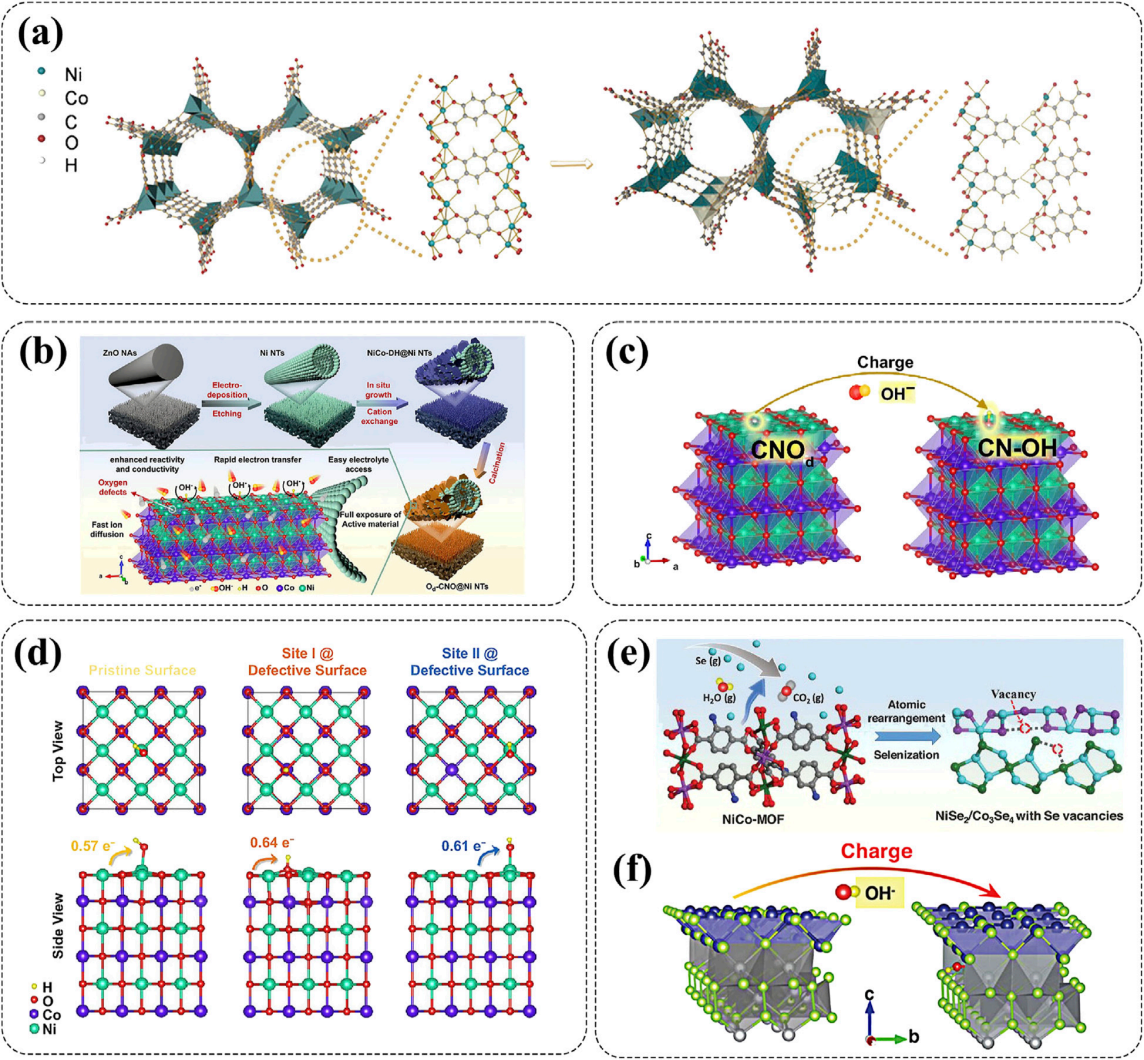
**(A)** Schematic diagram of the structural evolution from MOF-74(Ni) to MOF-74(NiCo) with designed dual-defects. Reprinted with permission from Chen T. et al. (2024). Copyright (2024) John Wiley and Sons. **(B–D)** Synthesis mechanism of O_d_-CNO@Ni NTs nanostructure, modulation mechanism model of oxygen defects on OH^−^ adsorption and surface charge transfer and OH^−^ adsorption energy analysis for O_d_-CNO@Ni NTs nanostructure. Reprinted with permission from Yao et al. (2021). Copyright (2021) Springer Nature. **(E, F)** The formation mechanism of vacancies in CNT-V-NiCoSe-400 and modulation mechanism model of Se vacancies on OH^−^ adsorption. Reprinted with permission from Li J. et al. (2024). Copyright (2024) John Wiley and Sons.

Defect engineering has gradually become a regarded rational strategy that can significantly modify the electronic properties, modulate lattice arrangement, and regulate crystal structures of Ni-based cathode materials from the inside out (Guo et al., 2022; Zhang et al., 2020; Xiong et al., 2020). According to the reaction mechanisms, introduced defects can be categorized into atomic vacancies, interstitial defect, substitutional defect, and higher-dimensional defects (Xiong et al., 2020). Among these, substitutional defect and atomic vacancies, such as oxygen vacancies, anionic vacancies, and cationic vacancies, have emerged as key strategies to improve the rate capability and stability of Ni-based cathodes. And, the presence of defects used as active sites can effectively promote the reaction kinetics of high-rate AZNBs (Zhang et al., 2020). For example, Chen and co-workers selected salicylic acid (SA) as the modulator to partially substitute 2,5-dihydroxyterephthalic acid (H_4_dobdc) in MOF-74(NiCo), leading to ligand deficiencies or coordination environment defects that resulted in the generation of additional oxygen vacancies (Figure 7A) (Chen T. et al., 2024). Under the synergistic effect of oxygen vacancies and Co-doping, the dual defects-engineered bimetallic MOF-74(Ni_0.675_Co_0.325_)-8 cathode (molar ratio of feeding SA/H_4_dobdc = 8) showcased a improved capacity of 218.6 mAh g^−1^ and energy density of 266.5 Wh kg^−1^ (17.22 kW kg^−1^) for AZNBs. Besides, Li et al. synthesized a Ni-Co oxide cathode (AM-O_v_-NCO/NF) with oxygen-rich vacancies, promoting the penetration of the electrolyte and improving its reaction activity for high capacity (322.8 mAh g^−1^ at 1 A g^−1^), remarkable cycle stability (91.9% capacity retention after 2000 cycles), and improved energy density (539.1 Wh kg^−1^) in AZNBs (Li X. et al., 2023).

Introducing defects into Ni-based nanostructures can also achieve high stability during the ion extraction/insertion and promote ions diffusion as well as electron transfer kinetics (Zhang et al., 2020). The further explanation is that the introduction of defects can change the lattice structure of cathode materials with enhanced stability, increased OH- adsorption sites, and improved conductivity along with modified Fermi energy levels simultaneously, for robust and high-capacity Zn-Ni batteries. For instance, Yao and co-workers introduced ultra-thin CoNiO_2_ nanosheets with plentiful oxygen defects (O_d_-CNO) on the surface of vertically arranged Ni nanotube arrays (Ni NTs), thereby obtaining Od-CNO@Ni NTs cathode material (Figure 7B) (Yao et al., 2021). Specifically, the introduction of oxygen defects could enhance the adsorption of OH^−^, contributing to an increase in charge transfer between the electrode and OH^−^ from 0.57 to 0.64 or 0.61 electrons for high-capacity (432.7 mAh g^-1^) and high-rate (218.3 mAh g^−1^ at 60 A g^−1^) Od-CNO@Ni NTs cathode (Figures 7C,D). Furthermore, the Od-CNO@Ni NTs//Zn battery demonstrated an exceptionally capacity retention of 93% after 5000 cycles, along with enhanced energy density of 547.5 Wh kg^-1^ at 92.9 kW kg^−1^. Similarly, the Co-doped Ni_3_S_2_ nanosheets with abundant sulfur vacancies as effective cathode materials for AZNBs were synthesized by Li and co-workers (Li X. et al., 2022). Thanks to rich sulfur vacancies, a lot of active sites for electrochemical reactions were created, thereby greatly improving specific capacity (183.9 mAh g^−1^ at 1 A g^−1^) and energy density (442.8 Wh kg^−1^). In addition to anion vacancies, cationic vacancies can also improve the capacity and rate performance of Ni-based cathode materials. Li et al. designed a CNT-V-NiCoSe-400 electrode with Se vacancies and illustrated the formation mechanism of Se vacancies (Figure 7E) (Li G. et al., 2024). Density functional theory (DFT) calculation confirmed that the existence of Se vacancies can improve the internal electron transfer and ion diffusion efficiency, and create more OH^−^ storage sites for the improved capacity (Figure 7F). As a result, the CNT-V-NiCoSe-400 electrode exhibited an excellent capacity of 384 mAh g^−1^ at 1 A g^−1^ and rate capability of 209 mAh g^−1^ at 150 A g^−1^. These results indicate that defect engineering can effectively regulate and realize multiple battery performance optimizations for advanced AZNBs.

### 4.2 Ligand engineering

**FIGURE 8:**
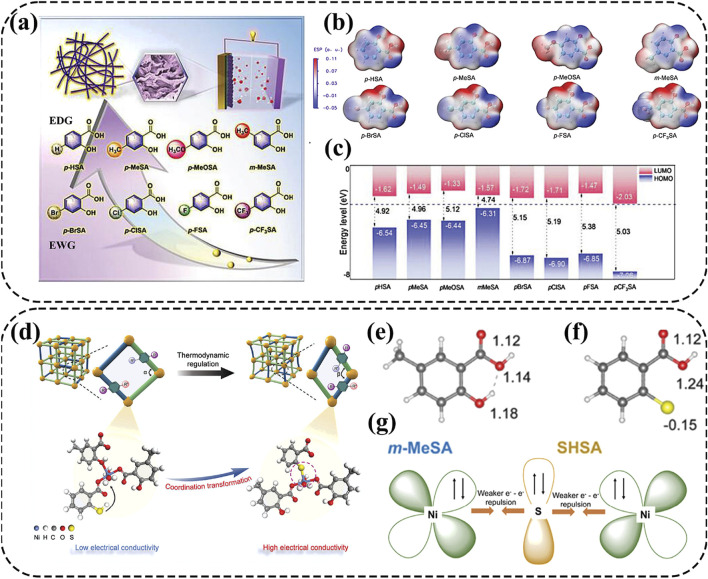
**(A–C)** Molecular structures of salicylic acid ligands containing different substituents, ESP distribution of SA with different substituents and frontier orbitals of SA with different substituents. Reprinted with permission from Su et al. (2023). Copyright (2023) John Wiley and Sons. **(D–G)** Schematic illustration of the synthetic process for NiSA-SSA-160 by thermodynamic modulation using a dual ligand-assisted strategy, the number of charges of the O atoms in the m-MeSA ligand, the number of charges of the O atoms and S atoms in the SHSA ligand and schematic representation of the electronic coupling to weaken e^-^-e^-^ repulsion and strengthen the coordination. Reprinted with permission from Su et al. (2024). Copyright (2024) John Wiley and Sons.

Metal-organic complex cathodes are expected to overcome the intermolecular forces and maintain good reversibility during charge-discharge cycles due to their remarkable chemical tunability and effective metal sites (Su et al., 2023; Su et al., 2024). For example, nickel-ligand coordination grids (NCGs) based on nickel and 3,5-pyridinedicarboxylic acid (3,5-PDC) ligands can form π-π stacks to facilitate electron transfer and boost structural flexibility for high-performance AZNBs (Zhang et al., 2021). Moreover, Su and co-workers chose salicylic acid (SA) as a ligand to synthesize a series of one-dimensional Ni-based SA complex cathode materials, which enhanced the electrical conductivity for improved battery performance of AZNBs (Figure 8A) (Su et al., 2023). Thereinto, the hydroxyl and carboxyl groups involved in the coordination of Ni-XSAs with electron-donating groups showcase a higher electron density around the ligands and decreased HOMO-LUMO gap, which were beneficial to coordination with Ni^2+^ and improved conductivity (Figures 8B,C). Consequently, the Ni-mMeSA cathode achieved a maximum energy density of 0.30 mWh cm^−2^ and a peak power density of 33.72 mW cm^−2^ for advanced AZNBs.

Although the study mentioned above provides a novel perspective to design Ni-organic complexes with enhanced conductivity, further improvements in electrochemical properties are necessary. To address this, Su et al. developed a Ni-based SA double-ligand complex by thermodynamic modulation using a dual ligand-assisted strategy (Figure 8D) (Su et al., 2024). Guest molecules containing sulfur induced competitive coordination with complexes involving Ni-O bonds, resulting in the S atom on the SHSA ligand acquiring a charge of −0.15. Therefore, the S atom was more easily coordinated with the transition metal to increase the electron density of the Ni center (Figures 8E,F). Furthermore, enhanced structural stability was confirmed through analysis of the electronic structure. Specifically, the introduction of thiols allowed the electrons of Ni^2+^ to coordinate with the orbitals of S, thereby weakening the e^-^-e^-^ repulsion, forming feedback bonds, and strengthening the coordination (Figure 8G). Consequently, the NiSA-SSA-160//Zn@CC battery achieved a capacity of 0.54 mAh cm^−2^ at 1 mA cm^−2^ with a maximum energy density of 0.54 mWh cm^−2^ at 49.5 mW cm^−2^, representing improved battery performance compared to the previous works. Therefore, ligand engineering can greatly improve the conductivity and reaction reversibility for high-rate and long-life AZNBs.

### 4.3 Ion doping

**FIGURE 9:**
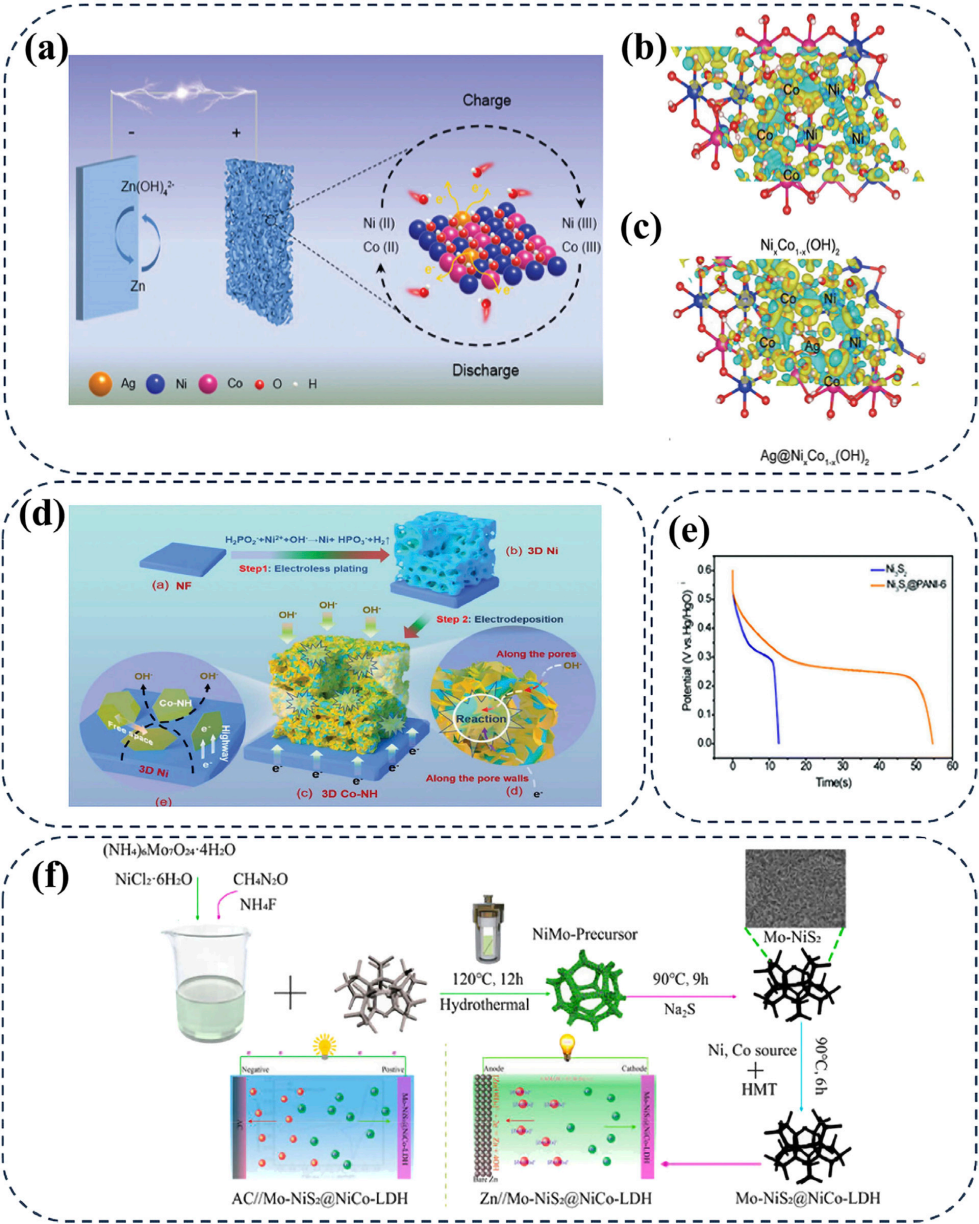
**(A–C)** The schematic diagram of the Ni–Zn battery based on Ni_x_Co_1-x_ (OH)_2_ doped with Ag atoms and the charge density differences on the Ni and Co site of Ni_x_Co_1-x_ (OH)_2_. Reprinted with permission from Tian et al. (2022). Copyright (2022) John Wiley and Sons. **(D)** The synthesis schematic of 3D porous Co-doping α-Ni(OH)_2_ nanosheets. Reprinted with permission from Wang K. et al. (2023). Copyright (2023) John Wiley and Sons. **(E)** Cycling stability at 17.1 A g^-1^ of Ni_3_S_2_ and Ni_3_S_2_@PANI-6 electrodes. Reprinted with permission from Zhou et al. (2019). Copyright (2019) John Wiley and Sons. **(F)** Schematic illustration for the stepwise construction of Mo-NiS@NiCo-LDH cathodes for high-performance asymmetric supercapacitor and aqueous Ni//Zn battery. Reprinted with permission from Shi et al. (2021). Copyright (2021) John Wiley and Sons.

To improve the stability of α-Ni(OH)_2_ in alkaline conditions and avoid its irreversibility between multiple phases, researchers have explored partial substitution of Ni^2+^ in α-Ni(OH)_2_ with heteroatoms (Co, Al, Zn, Cu, Mn, etc.) (Wei M. et al., 2018; Han et al., 2021). These heteroatoms can anchor anions (CO_3_^2-^, NO_3_^2-^, and OH^−^) and enhance the bonding force between Ni(OH)_2_ layers, thereby stabilizing the α-phase by strengthening the retention of anions and water molecules within the lattice. Simultaneously, the introduction of heteroatoms can effectively boost the catalyzed redox kinetic by modulating the charge distribution. For instance, Tian et al. developed Ag atom co-doping NixCo_1-x_ (OH)_2_ cathode via density functional theory (DFT) and unraveled the impact of atomic dopants on the electron structure in the Ni-based matrix (Figure 9A). The study revealed that atomic doping could modulate the charge distribution, as evidenced by the charge density differences at the Ni and Co sites in NixCo_1-x_ (OH)_2_, as shown in Figures 9B, C; (Tian et al., 2022).

Furthermore, in 3D macroporous α-Ni(OH)_2_ nanosheets (designed as 3D Co-NH), Wang et al. introduced Co^2+^ ions into the α-Ni(OH)_2_ nanosheets, creating abundant gaps that synergistically provide convenient and interconnected ion diffusion channels (Wang K. et al., 2023). These gaps also offer sufficient free space to accommodate volume changes of 3D Co-NH cathode during cycling, effectively improving the cycling stability of AZNBs after 1000 cycles at 6 mA cm^−2^ (Figure 9D). Accordingly, ion doping thus proves beneficial for designing cathode materials with high electrical conductivity and stability, particularly for those that typically suffer from irreversible phase transitions during charging and discharging. These transitions can be partially mitigated by structural distortions due to the isotropically distributed electrostatic interactions between metal ions. Compared to other Ni-based cathodes, Co-doped α-Ni(OH)_2_ exhibits an outstanding energy density of 543 Wh kg^-1^ and superior rate capacities at large current densities. Significantly, bimetallic co-doping has been proven to be an effective method for stabilizing α-Ni(OH)_2_. Recently, stable NiAlCo-LDH/CNT cathode has been reported to achieve a higher capacity of 354 mAh g^−1^ at 6.7 A g^−1^ and improved working voltage of 1.75 V than that of NiCo-LDH/CNT or AlCo-LDH/CNT cathodes (Gong et al., 2014). Therefore, ion doping can effectively modulate the electron structure and stabilize the structural robustness of the Ni-based cathodes for high-performance AZNBs.

### 4.4 Heterostructure regulation

Heterogeneous structures consist of regions with significantly different characteristics, and the interaction and coupling between these regions can produce synergistic effects (Zhu and Wu, 2023). By mixing or coating, the elegant introduction of additional active components (e.g., carbon-based materials (Yang et al., 2020), NiS (Zhou et al., 2020c), Co(OH)_2_ (Zhou K. et al., 2022), etc.) into target materials has become a recognized tactic to enhance electronic conductivity and electrochemical activity in energy storage fields. As expected, this approach has also been extended to the modification of Ni-based cathode materials in AZNBs (Li Y. et al., 2018). Currently, heterogeneous structures demonstrate great potential to enhance the capacity and long-term cycling stability of Ni-based cathode materials. For example, an polyaniline (PANI) surface coated Ni_3_S_2_ (Ni_3_S_2_@PANI) cathode exhibits a significantly longer discharge voltage platform compared to the pristine Ni_3_S_2_ electrode, as shown in Figure 9E (Zhou et al., 2019). This improvement confirms that PANI layer significantly enhances the capacity of the Ni_3_S_2_ electrode. Encouragingly, the Ni_3_S_2_@PANI electrode also exhibited significant durability without any capacity degradation after 10000 cycles.

Furthermore, heterostructured Ni-based composites can simultaneously utilize the advantages of two composite materials for high-performance AZNBs. Recently, Shi’s group adopted multistep hydrothermal method to synthesize the composite of the Mo-introduced nickel sulfide thin sheets decorated with Ni-Co layered double hydroxide nanosheet hierarchical heterostructures on Ni foam (denoted as Mo–NiS_2_@NiCo-LDH), as shown in Figure 9F (Shi et al., 2021). This heterostructure leverages the benefits of both Mo-NiS_2_ and NiCo-LDH to improve the redox active sites and stability of the Mo-NiS_2_@NiCo-LDH electrodes. Thus, Zn//Mo-NiS_2_@NiCo-LDH battery offers a high capacity of 275 mAh g^-1^ (1 A g^−1^) and reversible capacity of 182 mAh g^−1^ after 700 cycles (4 A g^−1^). Similar enhancements are observed in nickel hydroxide architectured, such as NiS-coated Ni(OH)_2_ cathode of AZNBs. Ni-based cathodes are well-known for suffering from insufficient reversible reactive sites, which makes it challenging to achieve high practical specific capacity and long cycle stability (Xu et al., 2016). To address this issue, Zeng et al. developed a Ni-NiO heterostructured nanosheet cathode to enhance the reaction kinetics and reversibility (Zeng et al., 2017). Specifically, the abundant embedded Ni nanoparticles can promote the electron transfer between the electroactive sites and the current collector during the battery cycling. Benefiting from the improved conductivity and electroactivity of the Ni-NiO heterostructured cathode, the assembled AZNBs shows unprecedented cyclic durability of 96.6% capacity retention after 10000 cycles. In summary, the heterostructure regulation strategy can increase electrochemical active sites and enhance electron transport capability of Ni-based cathodes, contributing to the high-energy and long-lifespan AZNBs.

### 4.5 Nanostructured optimization

**FIGURE 10:**
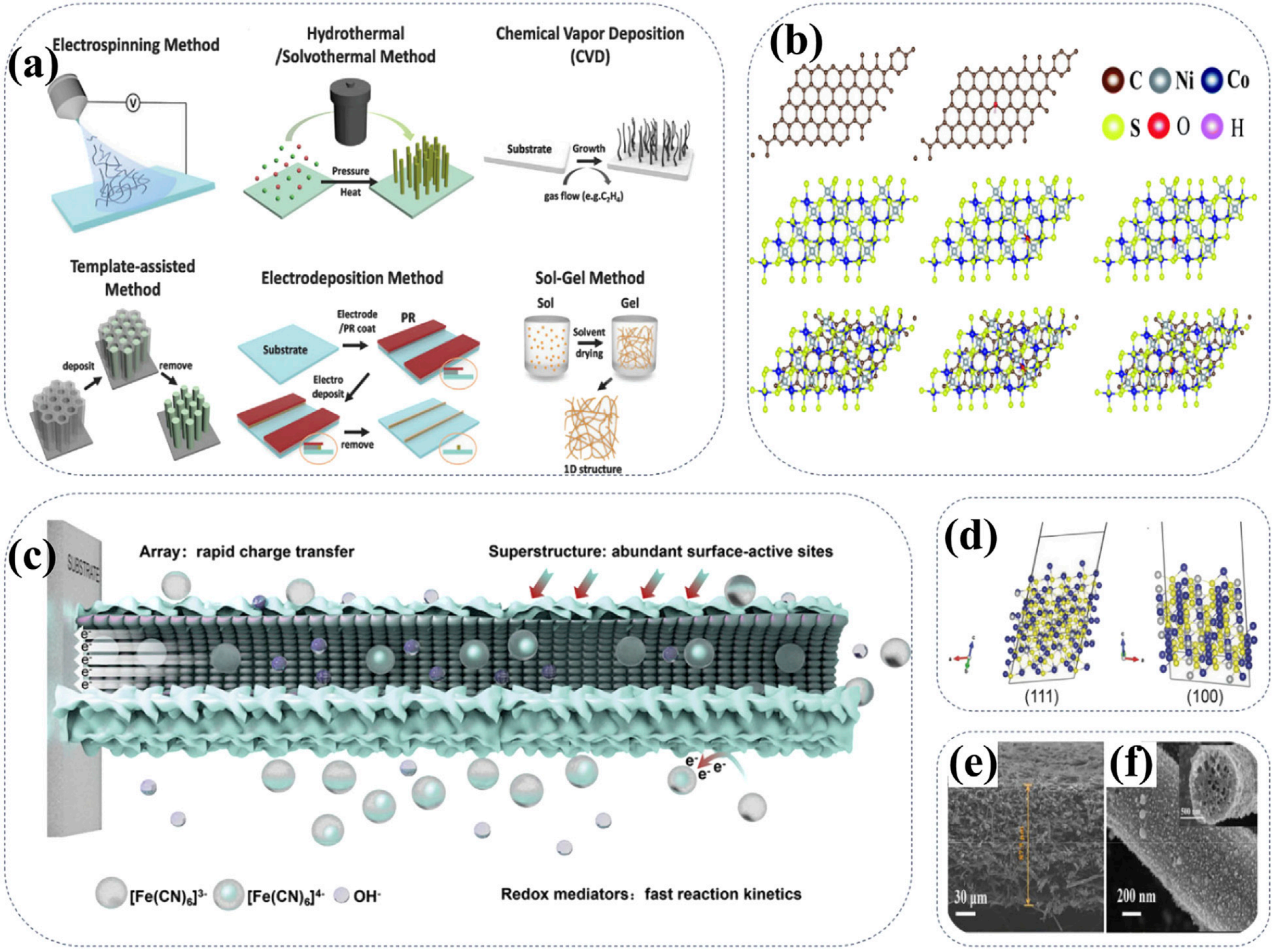
**(A)** Schematic illustration of typical synthetic methods for 1D nanostructures. Reprinted with permission from Cheong et al. (2022). Copyright (2022) John Wiley and Sons. **(B)** Models of the calculation of OH^−^ adsorption on carbon, NiCo_2_S_4_ and NiCo_2_S_4_/carbon. Reprinted with permission from Yu et al. (2022). Copyright (2022) Royal Society of Chemistry. **(C)** Illustration of the energy storage enhancement mechanism of CNSNA superstructure. Reprinted with permission from Zhang C. et al. (2024). Copyright (2024) John Wiley and Sons. **(D)** (111) and (100) surface of the NiCo_2_S_4_, respectively. The gray, blue, and yellow balls represent nickel, cobalt, and sulfur atoms, respectively. Reprinted with permission from Li W. et al. (2018). Copyright (2018) John Wiley and Sons. **(E, F)** SEM images of CNF@NiCo_2_S_4_. Reprinted with permission from Cui Z. et al. (2021). Copyright (2021) Elsevier.

As a cutting-edge technology, nanostructure optimization provides a new idea for improving the electrochemical performance of cathode materials for Ni-Zn batteries (Zuo et al., 2016; Cheong et al., 2022; Yu et al., 2022). Nanostructure optimization involves reducing the size of the cathode material to the nanoscale through nanoprocessing techniques, which can significantly increase the specific surface area, promote rapid charge transfer, and improve electrochemical activity. At the same time, controlling the morphology of materials (nanowires, nanosheets, nanoparticles, etc.) can further optimize electrochemical properties, such as enhancing structural stability and cycling stability. Typical methods for the synthesis of 1D nanostructures are shown in Figure 10A, including electrospinning, hydrothermal and solvothermal method, chemical vapor deposition, template-assisted approach, electrodeposition, and sol-gel method (Cheong et al., 2022). For instance, the NiCo_2_S_4_/HCS@CF film synthesized by electrospinning technology is lightweight, free of any polymer binders, thus exhibiting high conductivity, abundant electroactive sites, and favorable reaction kinetics for AZNBs. The DFT calculations confirm that the NiCo_2_S_4_/HCS@CFs have a high adsorption ability towards the OH^−^ ions in the electrolyte, thus greatly improving the reaction kinetics (Figure 10B) (Yu et al., 2022). In addition, two-dimensional (2D) materials have a sheet structure with nanometer thickness and microscale horizontal size, thus benefitting to the unique physicochemical properties, large surface area and abundant active sites for high-performance AZNBs. Very recently, with the introduction of two-dimensional materials, the performance of nickel-zinc batteries has also been improved (Lei et al., 2024).

The construction of heterostructures is an important way to optimize nanostructures. By constructing heterostructures of core-shell structures and nanocomposites, the properties of Ni-based cathode materials exhibit enhanced electron transport, structural stability and improved catalytic activity for the high-performance AZNBs (Zhang C. et al., 2024). For example, CoSe_2_@Ni_3_Se_4_@Ni(OH)_2_ superstructure nanoarray (CNSNA) material has been used as the cathode, which can greatly enhance the battery performance caused by the abundant active surface sites. In addition, the redox mediator K_3_ [Fe(CN)_6_] is used to accelerate the reaction kinetics by introducing a supplemental redox reaction, further improving charge storage capacity (Figure 10C) (Zhang C. et al., 2024). Moreover, the optimization of the nanostructure of cathode materials plays a crucial role in improving the ion transmission efficiency of AZNBs. At the atomistic scale, as shown in Figure 10D, NiCo_2_S_4_ cathode exhibits the minimum geometric diameters of 0.41 and 0.39 nm for (111) and (100), respectively (Li J. et al., 2018). These results provide the possibility of a large open channel for the overall diffusion of hydroxide ions (∼0.27 nm) into NiCo_2_S_4_, promoting the rapid ion diffusion tuunels for high-rate AZNBs during battery cycling. Figures 10E, F) show SEM images of carbon nanofibers functionalized with ternary NiCo_2_S_4_ nanoparticles (denoted as CNF@NiCo_2_S_4_), suggesting unique multi-channel structures for the high-performance CNF@NiCo_2_S_4_ cathodes (Cui Z. et al., 2021). Therefore, nanostructured optimization can effectively improve the electrochemical performance of Ni-based cathodes, improving its cycling stability and kinetic performance to meet the growing demand of advanced AZNBs.

## 5 Conclusion and outlook

**FIGURE 11:**
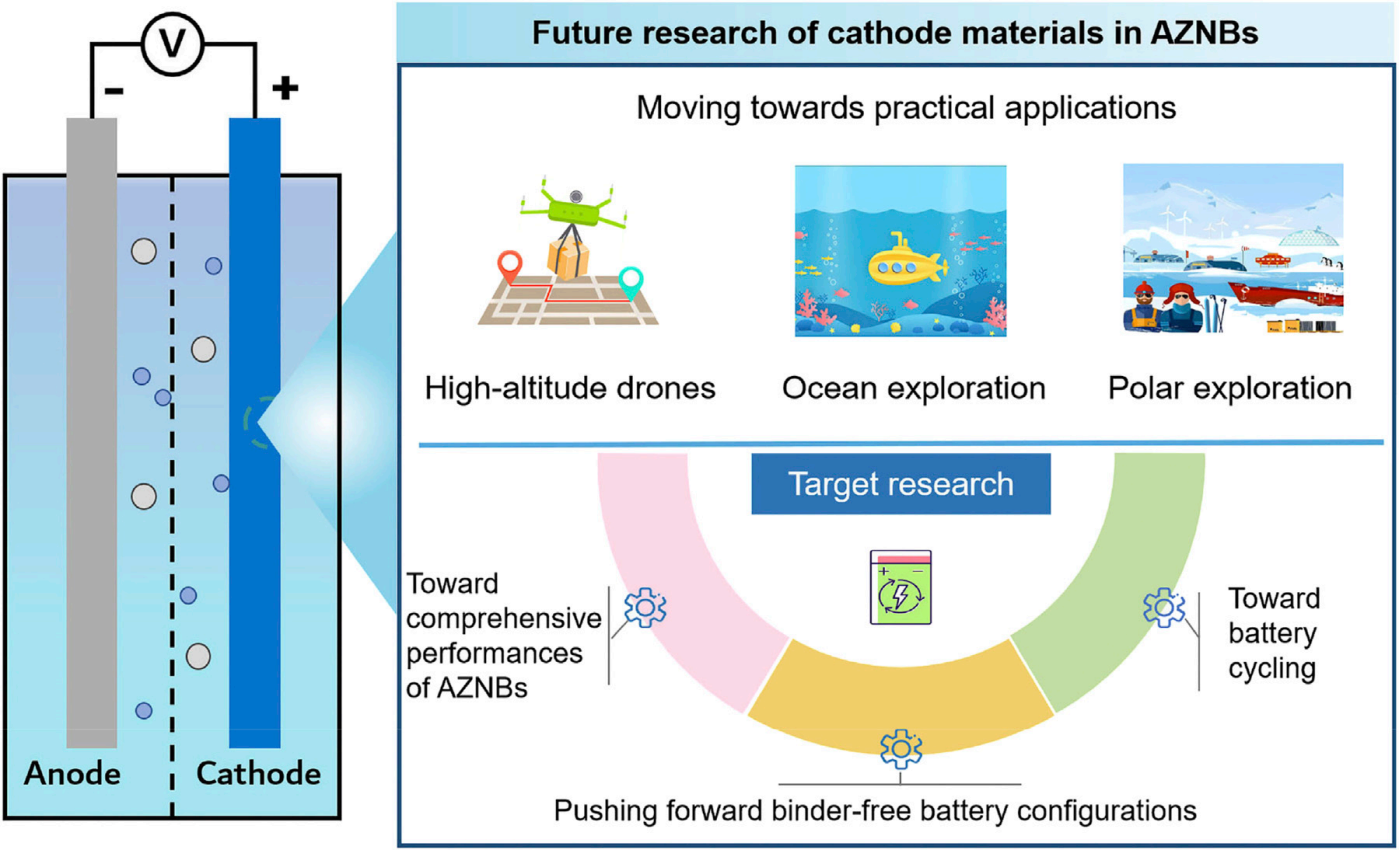
The future challenges and perspectives of Ni-based cathode materials for high-performance AZNBs.

In summary, aqueous Ni-based rechargeable batteries using Zn anode and an alkaline electrolyte have garnered increasing interests due to their intrinsic safety, environmentally friendliness, and high output voltage. This review began with an overview of the most recent progress and limitations of representative Ni-based cathode materials. We then systematically presented effective strategies to overcome obstacles and optimize electrochemical performance. While many valuable insights have been offered for designing Ni-based cathodes, AZNBs still require further advancements in both electrode and electrolyte development for practical applications. Key conclusions and perspectives are outlined below for the future development of advanced AZNBs, particularly focusing on research into Ni-based cathodes (Figure 11). Based on the high energy, long-lifespan, and robust targets, more strategies enabled binder-free electrodes, and improved comprehensive battery performance should be further explored to boost the practical application of advanced AZNBs. Table 1 summarizes the electrochemical performance of recently reported AZNBs based on Ni-based cathodes. Admittedly, still many achievements have been reported in this field to provide valuable directions for the future development of advanced AZNBs.

**TABLE 1:** Summary of cathodes, anodes, electrolytes, and the achieved battery perfornmances for recent reported AZNBs.

Cathode	Anode	Electrolyte	Capacity /mAh g^-1^	Energy density/Wh Kg^-1^	Capacity retention/%	Reference
Od-CNO@Ni	Zn	4 M KOH/2 M kF/1 M K_2_CO_3_/Sat. ZnO	334.9	547.5	93.0% 5000 cycles	Yao et al. (2021)
AM-O_v_-NCO /NF	Zn	3 M KOH/1% Zn(OAc)_2_	322.8	539.1	91.9% 2000 cycles	Li et al. (2023b)
CNT-V-NiCoSe	Zn	3 M KOH/Sat. ZnO	384	615.6	87% 5000 cycles	Li et al. (2024b)
CNMO-15	Zn	1 M KOH/0.01 M Zn(CH_3_COO)_2_	361.4	474.1	119.8% 5000 cycles	Shen et al. (2020)
Co-Ni_3_S_2–x_	Zn	3 M KOH/1% Zn(CH_3_COO)_2_	183.9	442.8	72.9% 6000 cycles	Li et al. (2022b)
M-LDH @MXene/NF	ZnO	6 M KOH/0.5 M ZnO/0.5wt%LiOH·H_2_O	311	465	88.6% 10000 cycles	Chen et al. (2019)
NiCo_2_S_4_/HCF@CFs	Zn plate	3 M KOH/0.02 M Zn(CH_3_COO)_2_	343.1	563.2	89.2% 1000 cycles	Yu et al. (2022)
NiFeCo-LDH	Zn	2 M KOH/0.2 M Zn(CH_3_COO)_2_	271.7	464.7	96% 15000 cycles	Wang et al. (2023a)
NF\Ni_3_S_2_/NiS@NiCo-LDH	Zn	6 M KOH/0.2 M Zn(CH_3_COO)_2_	317.9	556.3	116.7% 5000 cycles	Zhou et al. (2022a)
NiS-coated Ni_0.95_Zn_0.05_(OH)_2_	Zn mesh	4 M KOH/2 M kF/1 M K_2_CO_3_/Sat. ZnO	266.6	300	81.4% 800 cycles	Zhou et al. (2020c)
Ni_3_S_2_/PANI	Zn plate	6 M KOH/0.2 M Zn(CH_3_COO)_2_	242.8	386.7	100% 10000 cycles	Zhou et al. (2019)
3D Co-NH	Zn foil	7 M KOH/Sat. ZnO	284	534	76.9% 2000 cycles	Wang et al. (2023b)
G-NCGs	Zn plate	1 M KOH/20 mM Zn(CH_3_COO)_2_	113.8	189.2	50% 2000 cycles	Zhang et al. (2021)
NiS_2_/rGO	Zn sheet	1 M KOH/0.2 mM Z (CH_3_COO)_2_	209.4	357.7	80.5% 2000 cycles	Shi et al. (2019)

### 5.1 Pushing forward binder-free battery configurations

Binder-free AZNBs systems utilizing a uniform self-supported strategy based on Ni foam (NF) have been proposed, offering abundant reactive sites, rapid ion diffusion channels, and fast electron transfer pathways. The hierarchical structure design also provides an extensive active surface area for redox reactions (Cui Z. et al., 2021). Therefore, this configuration can achieve both high energy density and long lifespan for AZNBs. However, considering the cost-effectiveness of Ni/Co-based binder-free methods, future research should focus on developing economically efficient and straightforward strategies for synthesizing cathodes.

### 5.2 Toward comprehensive performances of AZNBs

Current research primarily focuses on improving the electrochemical performance of individual components, such as anodes, cathodes, electrolytes, in AZNBs. From the perspective of cathodes, greater emphasis should be placed on achieving high mass loading of active materials (>20 mg cm^−2^ in general) and low N/P ratios for commercial Zn-Ni batteries. Additionally, many newly optimized anodes in alkaline electrolytes are not yet commercially available due to the economic cost, environmental impacts, and power consumption. From the electrolyte perspective, lean alkaline electrolytes with low concentration should be stressed to decrease the self-corrosion and self-discharge of Zn anodes. Furthermore, OER at the cathode/electrolyte interface should be limited for high-efficient and robust AZNBs in large-scale energy storage applications (Zhou et al., 2023). Therefore, further research needed in electrode and electrolyte development for the large-scale energy storage applications of AZNBs.

### 5.3 Toward better battery recycling

As the world grapples with the consequences of the plastic crisis, it is imperative that we avoid replicating the same oversight with AZNBs that occurred with LIBs, where recycling was largely neglected during the early stages of commercialization. Given the potential of AZNBs as a sustainable alternative to LIBs, it is essential to prioritize research on their recycling processes, particularly in the context of large-scale deployment. Incorporating recycling efficiency into the design of electrodes and electrolytes is critical for enhancing the feasibility of effective recycling strategies. Consequently, the recycling of Zn-Ni batteries should be viewed not only as a challenge but also as a significant opportunity for future advancements in sustainable energy storage.

### 5.4 Moving towards practical applications

As energy storage systems are increasingly required to adapt to diverse practical application scenarios, higher requirements are being placed on the environmental adaptability of AZNBs. Thus, simulating actual application environments to explore the full battery performance of AZNBs under extreme conditions is a promising direction, especially for applications such as high-altitude drones, ocean exploration, and polar exploration. For instance, adding dimethyl sulfoxide (DMSO) into 1 M KOH can effectively decrease the lowest freezing pointe to −90°C, propelling the development of AZNBs under extreme low-temperature applications (Chen S. et al., 2022). However, the failure mechanism in alkaline battery systems under extreme conditions remain unclear, which hinders the establishment of corresponding measures for high-performance AZNBs. Therefore, investigating the performance and reliability of AZNBs in extreme conditions is of great significance for promoting the practical application of AZNBs.

In conclusion, AZNBs are regarded as promising alternatives to LIBs, owing to their relatively high power density, ease of encapsulation, and inherent safety. Through the persistent efforts of researchers and advancements in technology, significant progress has been made in the development of high-performance AZNBs. This review aims to provide comprehensive perspectives and insights into the development of Ni-based cathode materials. By further refining the anode, optimizing the cathode, and enhancing the coordination between the electrolyte, AZNBs have the potential to emerge as the next-generation of energy storage devices, even under extreme operating conditions.
